# Predicting mid-pelvic interspinous distance in women using height and pubic arch angle

**DOI:** 10.1371/journal.pone.0289814

**Published:** 2023-08-10

**Authors:** Eun Duc Na, Min Jung Baek, Ji Hyun Moon, Cho Won Park, Jin Yoon, Hanna Lee, So Hyeon Park, Ji Hyon Jang

**Affiliations:** Department of Obstetrics and Gynecology, CHA Bundang Medical Center, CHA University, Seongnam City, South Korea; Sohag University Faculty of Medicine, EGYPT

## Abstract

**Objective:**

To predict the interspinous distance (ISD) using the relationship between female height and pelvimetric measures on magnetic resonance (MR) images.

**Methods:**

We obtained measurements of the pubic arch angle (PAA), inlet-anteroposterior (AP) distance, mid-pelvis AP distance, outlet-AP distance, ISD, and ischial tuberosity distance using 710 pelvic MR images from nonpregnant reproductive-aged (21–50 years) women from January 2014 to June 2020. Patient height was also assessed from medical records. We determined the formula for predicting ISD using multiple regression analysis.

**Results:**

The mean ± standard deviation of the height, PAA, inlet-AP distance, mid-pelvis AP distance, outlet-AP distance, ISD, and ischial tuberosity distance were 160.0 ± 5.5 cm, 87.31 ± 6.6°, 129.7 ± 9.0 mm, 119.7 ± 8.5 mm, 111.71 ± 8.90 mm, 108.88 ± 8.0 mm, and 121.97 ± 11.8 mm, respectively. Two significant regression formulas for predicting ISD were identified as follows: ISD = 0.24973 × height − 0.06724 × inlet-AP distance + 0.12166 × outlet-AP distance + 0.29233 × ischial tuberosity distance + 0.32524 × PAA (*P* < 0.001, R^2^ = 0.9973 [adjusted R^2^ = 0.9973]) and ISD = 0.40935 × height + 0.49761 × PAA (*P* < 0.001, R^2^ = 0.9965 [adjusted R^2^ = 0.9965]).

**Conclusion:**

ISD is the best predictor of obstructed labor. This study predicted ISD with 99% explanatory power using only the height and PAA. The PAA can be measured by transperineal ultrasound. This formula may successfully predict vaginal delivery or cephalopelvic disproportion.

## Introduction

A successful vaginal delivery requires a well-matched maternal pelvis and fetal size plus presentation. Fetal size is measured by ultrasonography before the delivery. However, the maternal pelvis is not measured before labor, although labor per se has been reported as a way to measure the pelvis. Previous studies have examined the maternal pelvis using X-ray, computed tomography, and magnetic resonance imaging (MRI) [1–3]; however, the use of these methodologies is limited because of radiation exposure and high costs.

The maternal pelvis consists of the inlet pelvis, mid-pelvis, outlet pelvis, and pubic arch angle (PAA). The ischial spine is an important landmark in fetal engagement during labor. The station is zero when the fetal head descends to the level of this plane. The interspinous distance (ISD), i.e., the mid-pelvis, is the narrowest part of the pelvis [2], which may be commonly obstructed when labor occurs at this point. The ISD is a useful predictor of successful vaginal delivery [4]. To date, the ISD has been measured using the index and middle fingers [5], and its measurement requires substantial experience and clinical skills. Thus, cephalopelvic disproportion (CPD) at this point cannot be predicted accurately in advance.

A short stature is a risk factor for CPD, for example when maternal height is <145 cm [6], as women with short stature have smaller pelvic dimensions. Women who have delivered vaginally have a larger pelvic size than women with CPD [7]. Hence, we hypothesized that height affects female pelvic size, including the ISD, and that ISD-related relational expressions may be derived utilizing anatomical elements that affect the ISD but cannot be assessed directly [8]. This study aimed to determine the anatomical relationship between patient height and ISD plus other pelvic parameters obtained using MRI.

## Materials and methods

This study retrospectively analyzed the data of women who underwent pelvic MRI due to various reasons such as myomas, adenomyosis, and gynecological cancer from January 2014 to June 2020 at our hospital. Of the 750 women with MRI data, 710 women of reproductive age (20–50 years) were recruited. The exclusion criteria were congenital pelvic deformity and history of pelvic trauma.

Data on height and obstetric history were collected from medical records, and MRI were obtained from a picture archiving and communication system. Magnetic resonance pelvimetry was performed with the patient in the supine position, using three MRI systems (SIGNA 3.0T HDXT, GE Healthcare, Chicago, IL, USA, 2006; SIGNA ARCHITEC, GE Healthcare, 2018; and SIGNA HDX 1.5T, GE Healthcare, 2006).

**Fig 1:**
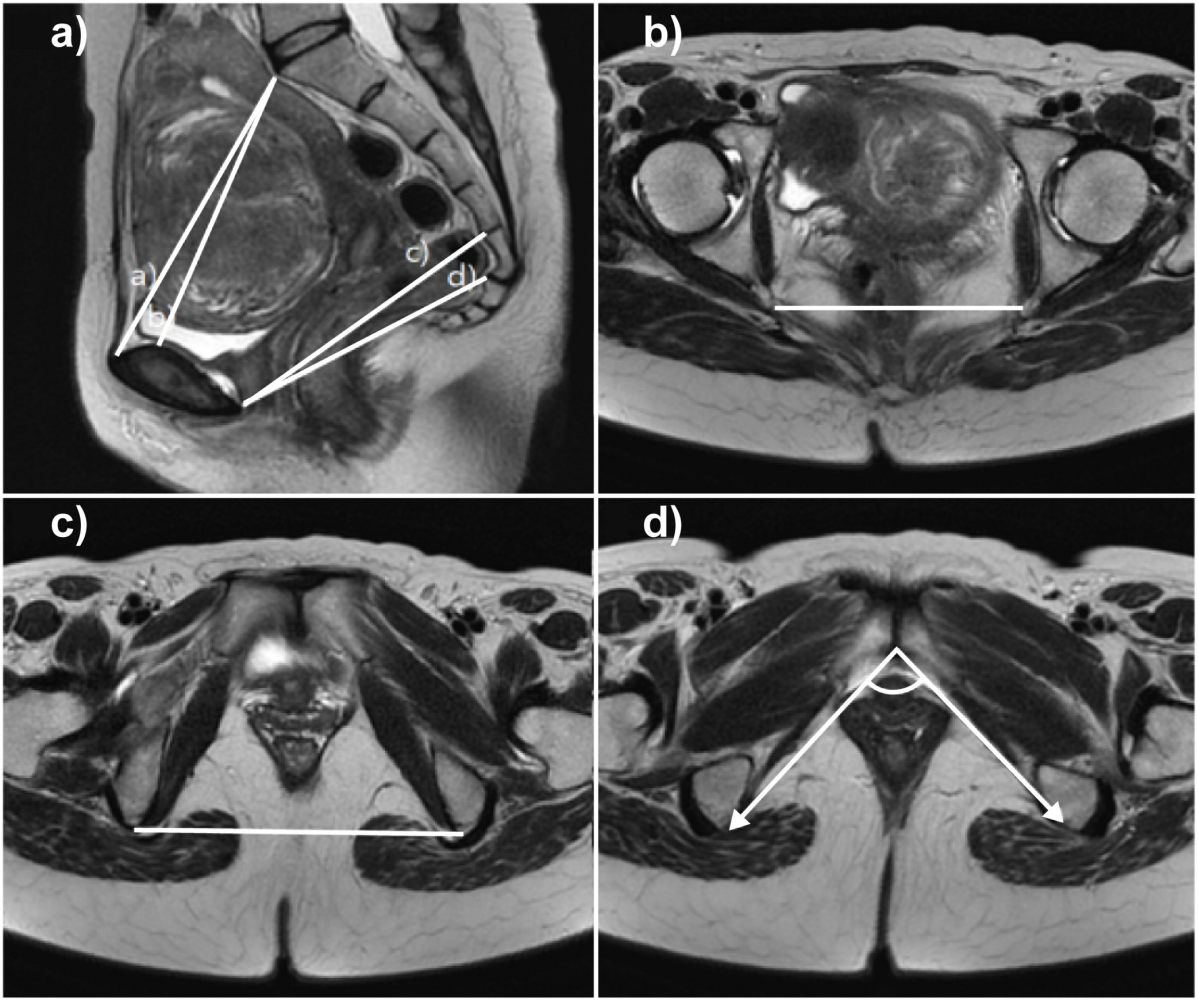
Pelvimetry parameters measured on magnetic resonance images of the female pelvis. (A) Sagittal view: (a) true conjugate (inlet-anteroposterior [AP] distance), (b) obstetric conjugate, (c) mid-AP distance, and (d) outlet-AP distance. (B) Interspinous distance in an axial plane at the level of the ischial spines. (C) Ischial tuberosity distance. (D) Pubic arch angle.

We measured the following seven parameters on MR images [4] (Table 1 and Fig 1): (1) true conjugate (inlet-anteroposterior [AP] distance), from the sacral promontory to the top of the symphysis pubis; (2) obstetric conjugate, from the sacral promontory to the inner margin of the symphysis pubis; (3) mid-AP distance, from the point between the S4 and S5 to the lower margin of the pubis symphysis; (4) outlet-AP distance, from the tip of the sacrum (not coccyx) to the bottom of the inner cortex of the symphysis pubis; (5) ISD (narrowest) between the ischial spines; (6) ischial tuberosity distance, the intertuberous (widest) distance between the ischial tuberosities; and (7) PAA, the angle between both ischial tuberosity and inter margin of the pubic symphysis (inferior).

**Table 1:** Landmarks of the pelvimetry parameters.

Parameter		Landmark	
Inlet	AP True conjugate (a)	Upper margin of the pubic symphysis	Promontory of the sacrum
	Obstetric conjugate (b) (shortest distance)	Inner margin of the pubic symphysis	Promontory of the sacrum
Mid	AP (c)	Lower margin of the pubic symphysis	The point between S4 and S5
	Transverse (interspinous distance)	Ischial spine distance	
Outlet	AP (d)	Lower margin of the pubic symphysis	Tip of the sacrum (not coccyx); lower border of S5
	Transverse	Ischial tuberosity distance	
Pubic arch angle		Inter margin of the pubic symphysis (inferior)	Ischial tuberosity

AP, Anteroposterior (distance).

R version 3.6.3 (R core team, 2020) was used for the statistical analysis, and statistical significance was set at *P* ≤ 0.05. The Shapiro–Wilk test and Quantile–Quantile plot were used to test for normality. Pearson correlation analyses were performed to determine the correlation between ISD and all other parameters. Two multiple linear regression analyses were performed. First, all parameters were used for measuring ISD. Second, the height and inlet-AP distance plus PAA, which were measurable by intrapartum ultrasound, for measuring ISD were analyzed. The minimal sample size was 153, as determined by a preliminary study using G*power version 3.1.9.4 [9], with an effective medium size of 0.15, a *P*-value of 0.05, a power of 0.95, and 7 predictors.

The study was approved by the Institutional Review Board and Ethics Committee of CHA Bundang Medical Center, CHA University, Seongnam City, South Korea (No. 2020-06-003, June 2, 2020). The requirement for informed consent was waived owing to the retrospective nature of the study.

## Results

Altogether, 710 women were included in this study. The mean ± standard deviation age of the patients was 40.5 ± 6.4 years (range, 21~50 years) and the mean height was 160.1 ± 5.5 cm. Patients’ mean inlet-AP distance, obstetric conjugate, mid-AP distance, outlet-AP distance, ISD, ischial tuberosity distance, and PAA was 129.7 ± 9.0 mm, 110.4 ± 8.1 mm, 119.7 ± 8.5 mm, 111.7 ± 8.9 mm, 108.9 ± 7.9 mm, 121.8 ± 11.7 mm, and 87.4 ± 6.6°, respectively. Regarding obstetric history, 245 women had a vaginal delivery, 146 women had a cesarean section, six women had only a stillbirth, and 313 women had no obstetric history (Table 2).

**Table 2:** Basic characteristics of patients.

	N = 750/710
Age (year)	40.5 ± 6.4
Height (cm)	160.1 ± 5.5
Weight (kg)	61.9 ± 12.3
Delivery mode	
NSVD	245
c/sec	146
None	313
Stillbirth	6
Inlet AP (cm)	129.7±9.0
Obstetric conjugate (mm)	110.5±8.1
Mid- AP distance(mm)	119.7±8.5
Outlet AP distance(mm)	111.7±8.9
Interspinous distance(mm)	108.9±7.9
Intertuberous distance (mm) (outlet trans)	121.8±11.7
Pubic angle (°)	87.4±6.6

NSVD, Normal spontaneous vaginal delivery; c/sec, Caesarean section; AP, Anteroposterior (distance); trans, Transverse (distance).

**Fig 2:**
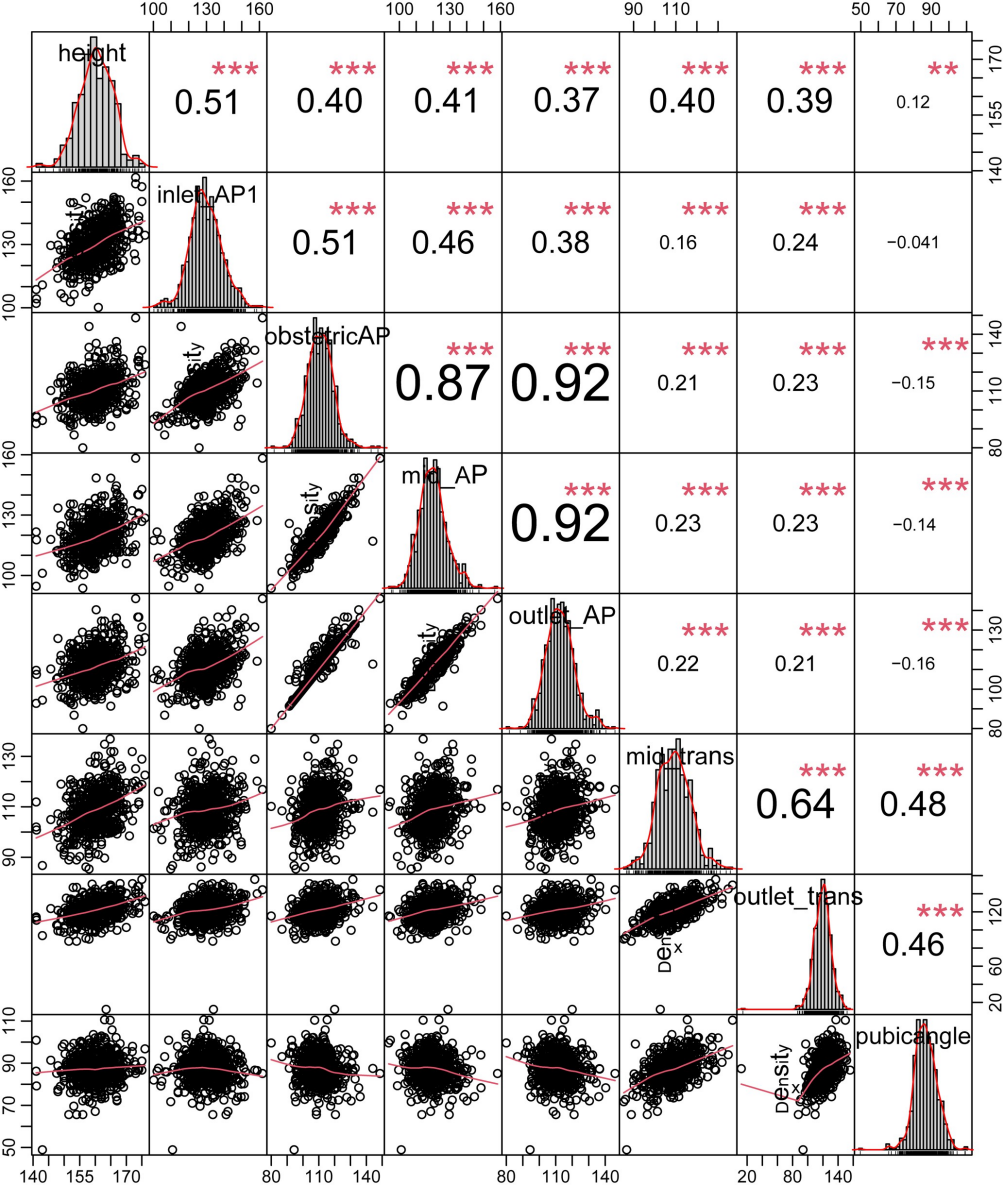
The distribution of all parameters. The scatterplots and Pearson correlation coefficient between each parameter.

The nonsignificant Shapiro–Wilk’s test revealed a normal distribution for the following parameters: inlet-AP distance, obstetric conjugate, mid-AP distance, outlet-AP distance, ISD, ischial tuberosity distance, PAA, and height (Fig 2).

All parameters and height were significantly linearly related to ISD. The Pearson correlation coefficients between ISD and each parameter are listed in Table 3.

**Table 3:** Pearson correlation coefficient between each parameter and interspinous distance.

Independent variable	Pearson correlation coefficient	*P-*value
Height	0.39	<0.001
Inlet-AP distance	0.16	<0.001
Obstetric conjugate	0.21	<0.001
Mid-AP distance	0.23	<0.001
Outlet-AP distance	0.22	<0.001
Ischial tuberosity distance	0.64	<0.001
Pubic arch angle	0.48	<0.001

AP, Anteroposterior.

The results of the multivariable linear regression models are presented in Tables 4 and 5. In the first model (Table 4), all parameters and height were used as independent variables. Stepwise linear regression was conducted, and the obstetric conjugate and mid-AP distance values were removed. The best model was ISD = 0.25268 × height − 0.07080 × inlet-AP distance + 0.12204 × outlet-AP distance + 0.29350 × ischial tuberosity distance + 0.32337 × PAA (R^2^ = 0.9973 [adjusted R^2^ = 0.9973], *P* < 0.001). The standardized coefficient was 0.37, −0.08, 0.13, 0.33, and 0.26 for height, inlet-AP distance, outlet-AP distance, ischial tuberosity distance (outlet transverse), and PAA, respectively. The most impactful factor was height. In this formula, R^2^ equals 0.9973, indicating a 99.73% explanatory power.

**Table 4:** The regression model using height and all parameters.

Dependent variable	Independent variable	Parameter estimate	Standard error	Standardized coefficient (β)	t	*P*-value	R^2^ (adjusted R^2^)	F	*P*-value
ISD	Height	0.25268	0.03421	0.37055	7.387	<0.001			
	Inlet-AP distance	−0.07080	0.02857	−0.08432	−0.2478	0.0134			
	Outlet-AP distance	0.12204	0.02730	0.12521	4.471	<0.001			
	Ischial tuberosity distance (outlet transverse)	0.29350	0.02209	0.32907	13.287	<0.001			
	Pubic arch angle	0.32337	0.03490	0.25953	9.265	<0.001			
**Model: ISD = 0.25268 × height– 0.07080 × inlet-AP distance + 0.12204 × outlet-AP distance + 0.29350 × ischial tuberosity distance + 0.32337 × pubic arch angle**							0.9973 (0.9973)	5.216e+04	<0.001

ISD, Interspinous distance; AP, Anteroposterior.

**Table 5:** The regression model using height and pubic arch angle.

Dependent variable	Independent variable	Parameter estimate	Standard error	Standardization coefficient (β)	t	*P*-value	R^2^ (adjusted R^2^)	F	*P*-value
ISD	Height	0.40935	0.01915	0.60029	21.38	<0.001			
	Pubic arch angle	0.49679	0.03498	0.39871	14.20	<0.001			
**Model: ISD = 0.40935 × height + 0.49679 × pubic arch angle**							0.9965 (0.9965)	9.985e+04	<0.001

ISD, Interspinous distance.

In the second model (Table 5), a multivariable linear regression analysis was performed using height and PAA plus the inlet-AP distance from intrapartum ultrasonography [8, 10]. The inlet-AP distance was not significant as a regression coefficient, and thus it was removed. The best model was fitted only using height and PAA (ISD = 0.40935 × height + 0.49679 × PAA (R^2^ = 0.9965 [adjusted R^2^ = 0.9965], *P* < 0.001). The standardized coefficient was 0.60 and 0.39 for height and PAA, respectively. In this formula, R^2^ equals 0.9965, indicating a 99.65% explanatory power.

## Discussion

ISD is a significant factor in CPD and is used as a criterion for fetal descent. This study attempted to investigate the anatomical relationship of ISD with other parameters, which is difficult to measure directly. Previous studies attempted to predict ISD using the PAA, although the explanatory power was only approximately 14% [8].

We measured women’s parameters according to reproductive age because the bone structure, which acts as a passage during delivery, does not change significantly during reproductive age [11].

Research has identified a connection between CPD and short maternal height [6, 12], with the majority of the studies based on the cut-off value of 150 cm or <145 cm [6].

Interestingly, maternal height was determined to be the most relevant factor in determining ISD in terms of anatomy in the current study. When comparing the standardization coefficients in the first model, the most determinate factor for ISD was confirmed to be height, rather than any other pelvic component. Hence, expecting that taller mothers will have fewer mid-pelvic contractions is reasonable.

The PAA is a factor that determines pelvic shape and differs significantly between the female and male pelvis. In general, the male pelvis has a small PAA, whereas the female pelvis has a large PAA. The android pelvis, also known as the male type of pelvis, is linked to CPD [13]. According to this study, because the PAA affects the ISD or the mid-pelvis, if a woman has a male-type pelvis, the PAA will be small, resulting in a short mid-pelvis. In the case of the android pelvis, it is the foundation for explaining its association with CPD.

In the FRABAT study, the PAA in women with vaginal breech delivery was not related to the success of vaginal breech delivery, although vaginal delivery was unsuccessful when the PAA was <70° [14]. Youssef et al. [15] and Ghi et al. [16] have reported that women who undergo caesarean delivery have a smaller PAA than those who undergo vaginal delivery, and that a permanent occipitoposterior positional relationship with a smaller PAA exists. Gilboa et al. [17] have also reported that a smaller PAA is related to a prolonged second phase of labor. These studies suggest a relationship between a small PAA and dystocia. It was directly confirmed through this study that PAA is an important factor in determining the ISD, which is an important factor in CPD.

A recent pelvic biometric study demonstrated that relaxin and estradiol enhance the interpubic space during pregnancy [18]. Pelvimetry is affected by changes in the relaxation of the surrounding ligaments as the pregnancy week develops [19]. Pelvic capacity is greater at 32 weeks than at 20 weeks; the inlet increases in the supine position, and the outlet increases in the semi-lithotomy position [20]. Therefore, the right posture and the frog pose during delivery are helpful. Dynamic external pelvimetry test results indicated that shifting position affects pelvic biomechanics [21]. When squatting, the outlet-AP distance increases to facilitate delivery. A technique for evaluating pelvic dimension using three dimensional MRI was recently presented [22]. This study did not investigate the effects of hormones that relax the pelvis, which limits the validity of the research and diminished its clinical relevance. Therefore, this study may be helpful in predicting mid-pelvis measurements before pregnancy.

The significance of the present study is that we obtained an anatomical relational formula with high explanatory power between ISD and other pelvimetry parameters, and it is especially meaningful that we obtained an ISD predictive formula with 99% explanatory power using only height and PAA. The results of this study can be thought of as hypothesis generation, and the results are preliminary and further investigation is needed to confirm validity and generalizability for the broader population.

When comparing the explanatory power of the equations, the first equation based on all parameters had an explanatory power of 99.73%, while the second equation based on factors that can be assessed by intrapartum ultrasonography had an explanatory power of 99.65%, indicating that both have a very high explanatory power. The strengths of this study are that the ISD value, which is generally difficult to measure, can be predicted through other pelvimetry parameters, and equations that can predict the ISD value with only ultrasonic measurable factors were obtained.

Because proper evaluation of the maternal pelvis during pregnancy is challenging owing to radiation concerns or expensive costs, it can be therapeutically beneficial for predicting CPD if the ISD can be predicted utilizing anatomical relational equations.

Nevertheless, this study had some limitations. First are the limitations and risk of bias that are inherent to the retrospective design. Second, The present study had the potential for selection bias. Third, This study is a study that found the anatomical relationship between pelvis and height in non-pregnant women. Therefore, considering our findings, it is necessary to conduct similar research on pregnant women to gain a more comprehensive understanding. Fourth, even if the ISD was predicted using a factor that can be measured by intrapartum sonography in the second equation, the position is different when MRI is utilized, and transperineal ultrasound for PAA measurement is not yet readily available. Finally, because the PAA is measured with the patient in the supine position during MRI, whereas that in intrapartum sonography is measured in a dorsal decubitus position with the legs ajar and semi-flexed [10], the PAA measured in MRI and the PAA measured in intrapartum sonography may be different [23].

Further research is needed to compare the pelvic measurements obtained via MRI with those of intrapartum sonography. The use of intrapartum pelvic ultrasound can even reflect the hormonal pelvic relaxation effect during delivery, which may be a cornerstone for studying CPD prediction. Additional validation is necessary to ensure that the study can be gnenralizaed to other groups and that the results can be subsequently applied to a broader population.

## Conclusion

In conclusion, this study predicts ISD values using pelvic bone and height data and demonstrates that the ISD can be predicted using the patient’s height and measured PAA. A comparison study of MR and ultrasound images will be required to predict ISD cutoff values in the delivery room. This should allow clinicians to anticipate CPD.
